# Geographic variation and effect of area-level poverty rate on colorectal cancer screening

**DOI:** 10.1186/1471-2458-8-358

**Published:** 2008-10-16

**Authors:** Min Lian, Mario Schootman, Shumei Yun

**Affiliations:** 1 Washington University School of Medicine, Department of Medicine, St. Louis, Missouri, USA; 2 Alvin J. Siteman Cancer Center at Barnes-Jewish Hospital and Washington University School of Medicine, St. Louis, Missouri, USA; 3 Missouri Department of Health and Senior Services, Division of Community and Public Health, Jefferson City, Missouri, USA

## Abstract

**Background:**

With a secular trend of increasing colorectal cancer (CRC) screening, concerns about disparities in CRC screening also have been rising. It is unclear if CRC screening varies geographically, if area-level poverty rate affects CRC screening, and if individual-level characteristics mediate the area-level effects on CRC screening.

**Methods:**

Using 2006 Missouri Behavioral Risk Factor Surveillance System (BRFSS) data, a multilevel study was conducted to examine geographic variation and the effect of area-level poverty rate on CRC screening use among persons age 50 or older. Individuals were nested within ZIP codes (ZIP5 areas), which in turn, were nested within aggregations of ZIP codes (ZIP3 areas). Six groups of individual-level covariates were considered as potential mediators.

**Results:**

An estimated 51.8% of Missourians aged 50 or older adhered to CRC screening recommendations. Nearly 15% of the total variation in CRC screening lay between ZIP5 areas. Persons residing in ZIP5 areas with ≥ 10% of poverty rate had lower odds of CRC screening use than those residing in ZIP5 areas with <10% poverty rate (unadjusted odds ratio [OR], 0.69; 95% confidence interval [95% CI], 0.58–0.81; adjusted OR, 0.81; 95% CI, 0.67–0.98). Persons who resided in ZIP3 areas with ≥ 20% poverty rate also had lower odds of following CRC screening guidelines than those residing in ZIP3 areas with <20% poverty rate (unadjusted OR, 0.66; 95% CI, 0.52–0.83; adjusted OR, 0.64; 95% CI, 0.50–0.83). Obesity, history of depression/anxiety and access to care were associated with CRC screening, but did not mediate the effect of area-level poverty on CRC screening.

**Conclusion:**

Large geographic variation of CRC screening exists in Missouri. Area-level poverty rate, independent of individual-level characteristics, is a significant predictor of CRC screening, but it only explains a small portion of the geographic heterogeneity of CRC screening. Individual-level factors we examined do not mediate the effect of the area-level poverty rate on CRC screening. Future studies should identify other area- and individual-level characteristics associated with CRC screening in Missouri.

## Background

Colorectal cancer (CRC) is the third most common cancer for both men and women in the United States, accounting for approximately 8–9% of both cancer incidence and mortality [1]. In 2008, there were an estimated 148,810 new CRC cases and an estimated 49,960 cancer patients will die of CRC nationwide [1]. CRC screening, including fecal occult blood test (FOBT), sigmoidoscopy or colonoscopy, reduces the risk of CRC death [2]. However, relative to breast cancer and cervical cancer, CRC screening use is still very low, but is increasing. The national breast cancer screening rate is about 70–80% [3], and the rate of cervical cancer screening is more than 80% [4,5]. Comparatively, the national CRC screening rate is much lower at about 50% [6]. Although there is an increasing secular trend in CRC screening rates across the United States [7], concerns about disparities in CRC screening have been rising in the past decade. Important disparities in CRC screening, which have been reported, include age [8,9], gender [8,10,11], race/ethnicity [11], obesity [12], individual socioeconomic status [8,13], education [14], availability and type of health insurance [11,13,15], and access to care [8,11,14]. However, it is still unknown if there is a geographic disparity in CRC screening associated with area characteristics.

Most previous studies mainly focused on individual-level risk factors of nonadherence to CRC screening use and have ignored area-level factors that have been found to be associated with breast and cervical cancer screening [5,16,17]. Multilevel studies may help examine the geographic variation, identify area-level characteristics that are associated with disparities in CRC screening, and provide evidence for targeting disadvantaged geographic areas to improve CRC screening. Based on our previous study which showed geographic variation in CRC screening across the United States [17], in this study we examined the geographic variation of CRC screening in our state (Missouri) and if area-level poverty rate was associated with CRC screening and substantially contributed to the geographic heterogeneity of CRC screening.

There are several potential mediating mechanisms by which higher area-level poverty rate may increase nonadherence to CRC screening guidelines. First, it may increase risk of nonadherence to CRC screening due to socio-demographic disparities (e.g., income, age) between areas [6]. Second, health conditions, including obesity, chronic disease morbidity (diabetes, asthma, cardiovascular diseases), and anxiety/depression symptoms, also may mediate the association between higher area-level poverty rate and CRC screening. Third, lack of access to medical care (e.g., health insurance coverage, personal primary care, routine medical check-up) may be an important reason for lack of CRC screening in high-poverty areas [11,14,18]. Fourth, lack of social support [19-22] in higher-poverty areas may help explain nonherence to CRC screening. Fifth, high area-level poverty rate may affect the CRC screening through health-related behaviors such as cigarette smoking, heavy alcohol consumption and physical inactivity. These behaviors have been reported more prevalent in areas with adverse conditions [23]. Sixth, the effect of area-level poverty rate on CRC screening may be mediated by self-rated health status. Many studies have indicated that adverse area conditions increase the risk of poor self-rated health [24-27].

In addition, area-level poverty rate may exert its influence on CRC screening through other factors which the BRFSS did not survey but were associated with CRC screening or other health behaviors/outcomes, such as cognitive and psychosocial factors (e.g., intention [19], optimism [19,28], self-efficacy/perceived behavioral control [19], knowledge about CRC [29], perceived benefits of CRC screening [19,20,30], perceived risk/susceptibility [10,21,28,30-32], stress [33], perceived racial discrimination [34] and perceived neighborhood safety/crime [35]), physician recommendation [20,21,32,36,37] and family history of CRC [19]. Availability and accessibility of health care (including safety-net clinics), characteristics of healthcare systems [8] and transportation [38] also may be important factors that could impact CRC screening in high-poverty areas.

In the current study, we determined if each or some of the six groups of individual-level factors from the BRFSS (socio-demographics, health conditions, access to medical care, social support, health-related behaviors and self-rated health status) accounted for any observed associations between area-level poverty rates and CRC screening.

## Methods

### Data Source and Design

Data from the 2006 Missouri BRFSS were used to assess the geographic variation and the effect of area-level poverty rate on CRC screening. The BRFSS is a survey of health risk factors and is sponsored by the Centers for Disease Control and Prevention (CDC). It is a standardized, random-digit-dialed telephone survey of the noninstitutionalized adult U.S. population carried out in all 50 states and the District of Columbia. In 2006, 5,391 Missourians age 18 or older were interviewed. The response rate of the 2006 Missouri BRFSS was 58.5%. To account for the complex sampling design, BRFSS data were weighted to adjust for the unequal probability of selection, differential nonresponse, and possible deficiencies in the sampling frame. We used a three-level design with individuals nested within self-reported five-digit ZIP code areas (ZIP5), which, in turn, were nested within three-digit ZIP code areas (ZIP3) that ignore the last two digits of ZIP codes.

In multilevel studies, a frequent concern is which geographic level should be considered [39] in order to target particular geographic areas. Inappropriate use of geographic levels can lead to the "Modifiable Areal Unit Problem" (MAUP) [40]. MAUP results from the use of artificial geographic units which were constructed from a continuous geographic area and may lead to the misinterpretation of results. A three-level multilevel design reduces the likelihood of MAUP bias to some degree [40].

The Institutional Review Board of the Missouri Department of Health and Senior Services (MDHSS) and Washington University reviewed the study protocol and determined it to be exempt. The original database provided by MDHSS did not contain identifiable information of study subjects.

### Colorectal Cancer Screening

According to the American Cancer Society's (ACS) guidelines [2], persons age 50 or older who are at average risk for CRC should have an annual fecal occult blood test (FOBT), a sigmoidoscopy every five years, or a colonoscopy every ten years. Because the BRFSS questions did not allow for the separation of colonoscopy and sigmoidoscopy use, we defined CRC screening adherence as having a FOBT in the past year and/or having a sigmoidoscopy/colonoscopy in the past five years for persons age 50 or older. We recognize that persons who reported having a colonoscopy 6–10 years prior to their interview would be misclassified as not adhering to ACS guidelines, thereby underestimating overall CRC screening rates. Because of the differences between FOBT and use of endoscopy (colonoscopy and sigmoidoscopy), we also performed the analysis separately for each test.

### Area-Level Poverty Rate

We used the percentage of the population living below the US federal poverty line from the 2000 census as a measure of area socioeconomic position. The poverty rate appears to be a measure that is robust across various diseases and levels of geography; it has policy implications, and is comparable over time [39].

We linked self-reported ZIP codes from the BRFSS to the 5-digit and 3-digit Zip Code Tabulate Areas (ZCTA5 and ZCTA3) from the 2000 census to obtain ZIP code-level poverty rates [41]. The ZCTA5 and ZCTA3, constructed from census blocks, are close approximations to 5-digit and 3-digit ZIP codes [42]. The ZCTA5- and ZCTA3-level poverty rates were categorized into less than 10%, 10–19% and 20% or higher to allow for nonlinear effects.

### Individual-level covariates

Data about the individual-level factors were obtained from the 2006 Missouri BRFSS and selected based on their associations with CRC screening [11,14,18-22]. We considered 18 individual-level covariates in six groups as potential factors by which area-level poverty would be associated with CRC screening (Table 1): (1) demographic characteristics (age, gender, race/ethnicity, marital status, education, employment status and family income); (2) personal health condition (body mass index [BMI], history of one or more health conditions [asthma, diabetes, heart attack, coronary heart disease, and stroke], and history of depression and/or anxiety); (3) access to medical care (health insurance coverage, having a primary care physician, and having a routine check-up); (4) social support; (5) health-related behaviors (current smoking status, alcohol use, and participation in physical activity); and (6) self-rated health status.

**Table 1:** Characteristics of the study population age 50 or older from the behavioral risk factor surveillance system, Missouri, 2006

**Variable**	**ZIP-5 level poverty rate**				**ZIP-3 level poverty rate**			
	<10%	10~19%	>= 20%	*P*	<10%	10~19%	>= 20%	*P*

Number of ZIP-5 areas	251	289	119		-	-	-	

Number of ZIP-3 areas	-	-	-		4	19	2	

Number of Participants*	1152	1346	489		586	2307	124	

†								

**CRC screening use**				<0.001				<0.001

No	43.5	51.5	51.4		42.3	49.1	60.3	

Yes	56.5	48.6	48.6		57.7	50.9	39.7	

**Endoscopy use**				<0.001				<0.001

No	49.7	57.9	57.4		47.6	55.6	65.8	

Yes	50.3	42.2	42.6		52.4	44.4	34.2	

**FOBT use**				0.118				0.753

No	84.8	85.8	88.8		85.7	85.9	88.2	

Yes	15.2	14.3	11.3		14.3	14.2	11.8	

**Demographics**								

Age				0.045				0.088

50–64 years	52.7	47.7	49.7		54.1	49.1	48.4	

65+ years	47.3	52.3	50.3		45.9	50.9	51.6	

Sex				0.891				0.126

Male	37.8	38.5	37.4		40.3	37.9	30.7	

Female	62.2	61.5	62.6		59.7	62.1	69.4	

Race/ethnicity				<0.001				<0.001

White	92.5	91.1	60.9		91.1	85.3	92.7	

African American	2.2	3.2	31.5		2.6	8.9	1.6	

Hispanic/Others	4.6	5.0	6.1		5.5	5.0	4.0	

Refused	0.7	0.7	1.4		0.9	0.8	1.6	

Marital Status				<0.001				<0.001

Married	57.0	52.8	38.9		61.1	50.2	47.6	

Not married	42.9	47.1	61.2		38.9	49.7	52.4	

Refused	0.1	0.1	0.0		0.0	0.1	0.0	

Education				<0.001				<0.001

≥ College-level	51.3	42.3	40.5		48.8	45.6	26.6	

<College-level	48.7	57.7	59.5		51.2	54.4	73.4	

Employment				0.003				<0.001

Employed	38.6	37.0	30.5		41.7	35.7	26.8	

Unemployed	17.3	19.0	24.8		14.5	20.2	30.1	

Retired	44.1	43.9	44.7		43.8	44.2	43.1	

Household income				<0.001				<0.001

>$50, 000	30.0	17.8	16.0		32.8	19.9	19.4	

$25,000 – 50,000	28.5	30.6	24.5		27.1	29.4	21.8	

<$25,000	27.3	38.0	47.7		28.5	36.4	50.0	

Missing	14.3	13.6	11.9		11.6	14.4	8.9	

**Health Condition**								

Body Mass Index				0.027				0.468

<25.0	31.6	31.4	26.2		28.7	31.3	26.6	

25.0 – 29.9	39.0	36.3	38.2		39.1	37.4	37.9	

30+	26.8	30.6	33.6		29.4	29.6	33.9	

Missing	2.6	1.6	1.8		2.9	1.8	1.6	

History of chronic Diseases				0.001				0.445

No	64.3	57.4	54.2		62.8	58.8	60.5	

One or more	34.9	41.8	45.0		36.5	40.3	39.5	

Don't know	0.8	0.9	0.8		0.7	0.9	0.0	

Anxiety/Depression				0.377				0.036

No	75.4	73.2	73.6		73.9	74.3	68.6	

Yes	20.1	20.5	20.5		22.0	19.6	28.2	

Don't know	4.5	6.3	5.9		4.1	6.1	3.2	

**Access to Care**								

Health insurance				<0.001				<0.001

Yes	94.3	92.3	88.3		96.6	91.4	91.1	

No	5.6	7.4	11.7		3.2	8.5	7.3	

Don't know	0.2	0.3	0.0		0.2	0.1	1.6	

Having a primary care physician			0.011					0.114

Yes	92.2	90.5	87.5		92.8	90.1	91.9	

No	7.8	9.5	12.5		7.2	9.9	8.1	

Routine check-up				0.001				

<1 yr	75.0	70.7	75.7		77.7	72.2	71.0	

2–5 yrs	17.4	16.6	13.9		16.2	16.5	16.9	

>5 yrs/Never	7.1	11.1	9.8		5.5	10.2	11.3	

Don't know	0.5	1.6	0.6		0.7	1.1	0.8	

**Social Support**								

Emotional support			<0.001					0.021

Always/Usually	78.2	74.2	65.0		79.2	73.1	72.6	

Sometimes/Never	17.2	19.4	28.8		16.4	20.8	24.2	

Missing	4.6	6.5	6.1		4.4	6.1	3.2	

**Health-related behavior**								

Smoking status				0.013				<0.001

Never	47.7	47.0	41.5		46.4	46.7	42.7	

Former	37.1	34.1	37.0		41.8	34.3	32.3	

Current Smoker	15.0	18.9	21.1		11.6	18.9	23.4	

Missing	0.2	0.1	0.4		0.2	0.1	1.6	

Heavy alcohol use				0.877				0.151

No	94.5	94.6	94.3		94.5	94.3	99.2	

Yes	3.9	3.4	3.7		4.1	3.6	0.8	

Missing	1.6	2.0	2.0		1.4	2.0	0.0	

Physically Active				<0.001				<0.001

Yes	71.3	63.7	62.0		71.2	65.9	52.4	

No	28.7	36.3	38.0		28.8	34.1	47.6	

**Health Status**								

Self-rated Health				<0.001				<0.001

Good/Above	75.4	67.7	62.4		75.5	68.8	60.5	

Fair/Poor	24.6	32.3	37.6		24.5	31.2	39.5	

* Participants with missing poverty rate were not listed; † values showed below are percentages.

Age was dichotomized as age 50–64 and 65 or older. Race/ethnicity was categorized as non-Hispanic white, non-Hispanic African American, and Hispanic/others. Marital status was grouped as married or not. Education level was categorized as college or higher level or less than college level. Employment status was categorized as employed, not employed, retired or refused. Household income was categorized in three groups: less than $25,000, $25,000–$50,000, and $50,000 or more. BMI was analyzed in three categories: <25.0, 25.0–29.9, and 30 or higher. Having ever been told by a doctor, nurse or other health professionals to have one or more of five chronic diseases, including diabetes, heart attack, angina or coronary heart disease, stroke and asthma, was considered as having a chronic disease history. Anxiety/depression history was defined as ever being told by a doctor or other health care providers to have an anxiety or depressive disorder. Having health insurance and having a primary care physician were both categorized as "yes" or "no." Routine medical check-up was categorized as "within one year," "two to five years," "more than five years" and "don't know." Always or usually needing emotional social support was compared to sometimes or never. We categorized smoking status as never smoked, former smoker, and current smoker based on responses to the following two questions: "Have you smoked at least 100 cigarettes in your entire life?" and "Do you now smoke cigarettes every day, some days, or not at all?" A never smoker was an individual who answered no to the first question. A current smoker was defined as an individual who responded yes to the first question and "every day" or "some days" to the second question. A former smoker is an individual who answered yes to the first question and "not at all" to the second question. Alcohol use was categorized as being a current heavy drinker or not. Heavy drinking was defined as having more than two drinks per day for men and having more than one drink per day for women. Persons who reported to have participated in physical activity or exercise during the past 30 days other than their regular job were considered as having been physically active. Self-rated health status was grouped into two categories: "good or better health" and "fair or poor health."

### Statistical Analysis

Data were analyzed using three-level logistic regression models. A total of 3,022 participants age 50 or older (Level one), were nested within 1036 ZIP5 areas (Level two), which, in turn, were nested within 25 ZIP3 areas (Level three). The complex sampling design in BRFSS was taken into account in the analysis. Models were weighted by a normed weight on the basis of the final weight variable that was calculated by the CDC. This normed weight was the multiplication of the BRFSS-final-weight by the ratio of participants age 50 or older in Missouri [43]. All models were fitted by second order penalized quasi-likelihood (PQL) estimation using the *MLwiN *2.02 software [44]. We found no evidence of extra binomial variation using a chi-square test in an empty model without any covariates or poverty rate, suggesting that the logistic model was appropriate. Fixed effects of poverty rate and random effects of CRC screening use across ZIP5 and ZIP3 were estimated for all models. To quantify the random effect of CRC screening, random intercept models were constructed in all analyses. The deviance information criterion (DIC) was used to evaluate the model fit. Smaller DIC values mean better model fit. Intraclass correlations (ICC) were computed to estimate the proportion of geographic variation across the ZIP5 and ZIP3 areas of the total variation, including variation across and within the ZIP5 and ZIP3 areas. The variation at level one was fixed at *π*^2^/3 (approximately 3.29) [45]. Because of the limitation of the ICC for binary response variables when measuring geographic variations and comparing fixed effects of area-level variables, we applied the methodology developed by Larsen and colleagues to compute the median odds ratio (MOR) and the 80% interval odds ratio (IOR) [46]. The MOR was calculated to quantify the variation of CRC screening across ZIP5s and ZIP3s using the equation: $MOR=\exp\left(Z_{0.75}\times \sqrt{2\times Var}\right)$. Where Var is the variance at ZIP5- or ZIP3-level, and Z_0.75 _is the value of standard normal distribution at 75% percentile (equals 0.6745). The "exp" is the exponential function. The MOR is always equal to or greater than one, and a larger MOR value indicates a larger geographic variation.

The IOR was calculated to quantify the fixed effect of area poverty rate using equations: $IOR_{UPPER}=\exp\left(\beta +Z_{0.90}\times \sqrt{2\times Var}\right)$ and $IOR_{Lower}=\exp\left(\beta +Z_{0.10}\times \sqrt{2\times Var}\right)$. Where *β *is the regression coefficients of area-level poverty rate, Z_0.90 _and Z_0.10 _are the values of standard normal distribution at 90% and 10% percentiles, respectively (equals ± 1.2816) and Var is the variance at the corresponding ZIP-level poverty rate. The range of IOR values also reflects the degree of variation between areas. A substantial contribution of the poverty rate to the geographic variation of CRC screening is present when the IOR does not contain the value of one.

To examine the associations between individual-level factors and CRC screening, a multilevel logistic regression analysis, involving all individual-level factors and area-level poverty, was used to estimate adjusted odds ratios and their 95% confidence intervals. Then, we constructed nine models to examine the role of area-level poverty rate on CRC screening. First, an empty model without any fixed effects was constructed to calculate the MORs at level 2 and 3. Second, we determined the unadjusted association of ZIP5- and ZIP3-level poverty rate with CRC screening use. Third, we assessed the potential mediating effect of each of the six hypothesized pathways on the association between ZIP5- and ZIP3-level poverty rates and CRC screening by constructing a separate model which included a set of individual variable and ZIP5- and ZIP3-level poverty rates. Finally we also fitted a model with ZIP5- and ZIP3-level poverty rates and all individual-level variables. Reduction in odds ratios and MOR for the ZIP5- and ZIP3-level poverty rate relative to unadjusted analysis was used as evidence for mediation. We also reran the analyses with only individual-level covariates significantly associated with CRC to reduce the likelihood of collinearity among these covariates.

## Results

The 2006 CRC screening rate among persons aged 50 and older in Missouri was 51.8% (endoscopy use: 45.5%; FOBT use: 14.1%). Table 1 shows the characteristics of the study participants. CRC screening use and endoscopy use, but not FOBT use, were lower in ZIP5 and ZIP3 areas with higher poverty rates. Similarly, a lower percentage of the population in ZIP5 and ZIP3 areas with higher poverty rates reported being married, having at least a college-level education, being employed, having a higher family income, and being in good health. Conversely, there were a higher proportion of smokers and those with BMI ≥ 30.0 in ZIP5 and ZIP3 areas with higher poverty rates. Additionally, a higher percentage of persons who reported being African American, having a history of chronic diseases, no health insurance plan, and no personal doctor when needed, resided within the higher-poverty ZIP5 areas, but not within the higher-poverty ZIP3 areas.

### Associations between individual characteristics and CRC screening within the same areas

The multivariable multilevel models adjusted for all individual covariates and area-level poverty rate showed that people who were retired, with higher BMI and anxiety/depression history had higher odds of CRC screening (Table 2). In contrast, non-African American minorities, people with a lower education level and lack of access to care had lower odds of CRC screening. People with mid-low household income ($25,000 to 50,000) also had slightly lower odds of having CRC screening. The strongest individual-level indicator for being non-adherent to CRC screening is lack of access to medical care specifically lack of health insurance coverage (OR = 0.57, 95% CI: 0.35–0.90), having no primary care physician (OR = 0.48, 95% CI: 0.32–0.74) and not having a routine check-up (2–5 yrs: OR = 0.41, 95% CI: 0.32–0.54; >5 yrs: OR = 0.12, 95% CI: 0.05–0.27).

**Table 2:** Adjusted fixed effects of individual-level factors on colorectal cancer screening in Missouri, 2006.

Variable	Odds Ratio (95% Confidence Interval)*		
	CRC screening	Endoscopy use	FOBT use

**Demographics**			

Age (vs. 50–64)			

65+	1.18 (0.90–1.55)	1.33 (1.03–1.72)	1.50 (0.80–2.82)

Sex (vs. Male)			

Female	0.87 (0.64–1.17)	0.89 (0.68–1.16)	0.88 (0.61–1.28)

Race/ethnicity (vs. White)			

African American	1.47 (0.98–2.20)	1.64 (1.06–2.52)	0.75 (0.21–2.68)

Hispanic/Others	0.56 (0.43–0.72)	0.49 (0.34–0.72)	1.03 (0.44–2.41)

Marital Status (vs. Yes)			

No	0.84 (0.59–1.21)	0.81 (0.55–1.20)	1.13 (0.69–1.86)

Education (vs. ≥ College-level)			

<College-level	0.70 (0.52–0.95)	0.69 (0.49–0.98)	0.89 (0.64–1.24)

Employment (vs. Employed)			

Not	1.17 (0.85–1.61)	0.97 (0.71–1.33)	1.25 (0.68–2.30)

Retired	1.43 (1.15–1.79)	1.16 (0.92–1.46)	1.02 (0.55–1.90)

Income (vs. >$50 k)			

$25 k~50 k	0.78 (0.62–0.98)	0.80 (0.66–0.98)	0.58 (0.34–1.01)

<$25 k	0.85 (0.67–1.08)	0.86 (0.63–1.18)	0.78 (0.50–1.22)

**Health Condition**			

BMI (vs. Normal)			

Overweight	1.40 (1.04–1.88)	1.30 (1.00–1.69)	1.12 (0.67–1.87)

Obesity	1.57 (1.13–2.18)	1.45 (1.07–1.98)	1.04 (0.67–1.62)

Comorbidity (vs. No)			

No			

Yes	0.96 (0.71–1.31)	1.06 (0.82–1.38)	1.00 (0.61–1.65)

Mental History (vs. No)			

Yes	1.44 (1.14–1.83)	1.70 (1.44–2.01)	0.78 (0.49–1.22)

**Access to Care**			

HealthPlan (vs. Yes)			

No	0.57 (0.35–0.90)	0.58 (0.38–0.89)	0.56 (0.25–1.25)

Personal Doctor (vs. Yes)			

No	0.48 (0.32–0.74)	0.42 (0.27–0.64)	0.67 (0.25–1.81)

Check-up (vs. <1 yr)			

2–5 yr	0.41 (0.32–0.54)	0.55 (0.41–0.75)	0.24 (0.13–0.46)

>5 yr/Never	0.12 (0.05–0.27)	0.13 (0.06–0.25)	0.19 (0.05–0.82)

**Social Support**			

Emotional support need (vs. Usually+)			

Sometimes/rarely/Never	0.78 (0.58–1.06)	0.83 (0.63–1.10)	1.12 (0.85–1.46)

**Health-related behavior**			

Smoking (vs. Never)			

Former	1.01 (0.62–1.64)	0.96 (0.66–1.39)	1.33 (0.86–2.07)

Somedays/EveryDay	0.89 (0.65–1.22)	0.82 (0.63–1.07)	1.28 (0.64–2.55)

Heavy Drinker (vs. No)			

Yes	1.73 (0.89–3.38)	2.20 (1.09–4.44)	0.78 (0.22–2.76)

Physical Exercise (vs. Had)			

No	0.90 (0.68–1.17)	0.89 (0.68–1.16)	0.86 (0.60–1.22)

**Health Status**			

Self-rated Health (vs. Good+)			

Fair/Poor	1.00 (0.79–1.26)	1.06 (0.82–1.36)	0.88 (0.62–1.26)

* All of three 3-level multilevel logistic models were adjusted for all individual-level factors and poverty rates at ZIP5- and ZIP3-level.

Table 2 also shows differences in the associations of the individual covariates with FOBT and endoscopy use. While older Missourians, African Americans, those overweight or obese, those with a history of anxiety/depression, and those considered to be heavy drinkers were more likely to report having had an endoscopy in the past five years; non-African American minorities, persons with a high school or lower education, and those without health insurance coverage or a primary care physician were less likely to report having had an endoscopy within the past five years. However, none of these covariates were associated with FOBT use in the past year. Only Missourians without a routine check-up were significantly less likely than those with a routine checkup to report having FOBT use in the past year.

### Area-level poverty rate and CRC screening

At the contextual level, increased second- and third-level area poverty rates were associated with lower CRC screening (Table 3). All nine models indicated significant geographic variation across ZIP5 areas, but not across ZIP3 areas. All nine ZIP5-level MOR values were similar (about 2.0), suggesting that the geographic variation was relatively large and indicating that, for subjects with the same individual covariates, persons living in areas with high poverty rates had about two times of median odds of not following CRC screening guidelines compared with persons living in areas with lower poverty rates. The MOR is related to the fixed-effects odds ratios (OR) for area-level variables. While the description of the adjusted OR of an area-level variable is fixed across the geographic areas, the MOR describes the variation across the geographic areas accounting for all area- and individual-level characteristics in the multivariable model. More details about the MOR are described by Larsen and Merlo [46].

**Table 3:** Association between CRC screening use and poverty rate at ZIP5 and ZIP3 level by examining six potential pathways.

	Model I	Model II	Model III	Model IV	Model V	Model VI	Model VII	Model VIII	Model IX
**Fixed Effects**									

**ZIP5 poverty **(>= 10% vs. <10%)	-	0.69 (0.58–0.81)	0.72 (0.62–0.85)	0.67 (0.57–0.79)	0.77 (0.66–0.90)	0.70 (0.59–0.82)	0.71 (0.61–0.83)	0.69 (0.59–0.81)	0.81 (0.67–0.98)

IOR *	-	0.18–2.62	0.19–2.71	0.17–2.60	0.21–2.88	0.18–2.65	0.19–2.67	0.18–2.62	0.22–3.00

**ZIP3 poverty **(>= 20% vs. <20%)	-	0.66 (0.52–0.83)	0.74 (0.55–0.98)	0.64 (0.51–0.81)	0.62 (0.51–0.76)	0.66 (0.52–0.83)	0.69 (0.52–0.91)	0.66 (0.52–0.83)	0.64 (0.50–0.83)

IOR	-	-	-	-	-	-	-	-	-

**Random Effects**									

**ZIP5 **[Var. (SE)]	0.573 (0.167)	0.543 (0.139)	0.529 (0.131)	0.558 (0.143)	0.529 (0.145)	0.546 (0.139)	0.534 (0.138)	0.544 (0.139)	0.519 (0.127)

MOR^†^	2.06	2.02	2.00	2.04	2.00	2.02	2.01	2.02	1.99

ICC (%)	14.83	14.17	13.85	14.50	13.85	14.19	13.96	14.19	13.63

PCV^‡ ^(%)	**-**	-5.24	-7.68	-2.62	-7.68	-5.06	-6.81	-5.06	-9.42

**ZIP3 **[Var. (SE)]	0.061 (0.034)	0	0	0	0.003 (0.018)	0	0	0	0

MOR	1.27	1.00	1.00	1.00	1.05	1.00	1.00	1.00	1.00

ICC (%)	1.82	0	0	0	0.09	0	0	0	0

**ZIP3 & ZIP5**	0.634	0.543	0.529	0.558	0.532	0.544	0.534	0.544	0.519

MOR	2.14	2.02	2.00	2.04	2.01	2.02	2.01	2.02	1.99

**DIC**^§^	4226	4163	4060	4149	3699	4152	4128	4155	3621

Note: Model I is an empty model; Model II only contains area poverty; Model III contains area poverty and demographic variable (age, gender, age, race/ethnicity, marital status, education, employment and income); Model IV contains area poverty and health condition variables (BMI < weight/height^2^>, chronic diseases history <asthma, diabetes, stroke, coronary heart disease and heart attack> and depression/anxiety history); Model V contains area poverty and access to care variables (health plan coverage, primary care physician and routine check-up for medical status); Model VI contains area poverty and emotional support; Model VII contains area poverty and health-related behaviors (smoking, alcohol-consumption and physical exercise); Model VIII contains area poverty and self-rated health status; Model IX contains area poverty and all individual-level factors. * IOR: Interval odds ratio; ^† ^MOR: Median odds ratio; ^‡ ^PCV: Proportional change in variance relative to Model I; §DIC, Deviance information criterion; ^¶ ^*P *< 0.01.

The empty model showed that 14.8 percent of the total variation in CRC screening lay between ZIP5 areas (Model I). The level-3 variation between ZIP3 areas was negligible (<2.0%, *P *> 0.05) in the multilevel logistic model. Because no difference in CRC screening was observed for persons residing in ZIP5 areas with poverty rates of 10–19% and 20% or higher we combined both categories. This was also the case for persons who resided in ZIP3 areas with poverty rates of <10% and 10–19%, which were also combined into one category. When only ZIP5 and ZIP3 poverty rates were included in the model, (Model II), the odds ratios for poverty rates greater than 10% was 0.69 for ZIP5 areas and 0.66 for ZIP3 areas with poverty rates of 20% or higher. When adjusted for the six sets of individual-level potential pathways separately (Models III – VIII), the odds ratios for poverty rate ranged from 0.67 to 0.81 for ZIP5 areas and from 0.62 to 0.74 for ZIP3 areas. With all 18 individual-level covariates in the model (Model IX), odds ratios were 0.81 (95% CI: 0.67 – 0.98) and 0.64 (95% CI: 0.50 – 0.83), respectively, for ZIP3 and ZIP5. Sensitivity analysis showed that the association was robust and the significance of the association between area-level poverty rate and CRC screening was never altered when including only variables associated with CRC screening in the model. IORs of ZIP5-level poverty rate ranged from 0.17–3.00 and were not significantly altered from model I to model IX.

The results for CRC screening closely followed the results for endoscopy use (Table 4). However, results were different for FOBT use (Table 5). There was no association between area-level poverty rate and FOBT use within the past year. The geographic variation in FOBT use was substantially larger across ZIP5 areas than for endoscopy use.

**Table 4:** Association between endoscopy use and poverty rate at ZIP5 and ZIP3 level by examining six potential pathways.

	Model I	Model II	Model III	Model IV	Model V	Model VI	Model VII	Model VIII	Model IX
**Fixed Effects**									

**ZIP5 poverty **(>= 10% vs. <10%)	-	0.66 (0.54–0.79)	0.66 (0.55–0.79)	0.64 (0.52–0.78)	0.70 (0.58–0.85)	0.66 (0.54–0.80)	0.68 (0.56–0.81)	0.66 (0.54–0.80)	0.72 (0.58–0.90)

IOR *	-	0.18–2.38	0.19–2.28	0.17–2.35	0.20–2.49	0.18–2.38	0.19–2.42	0.18–2.38	0.21–2.48

**ZIP3 poverty **(>= 20% vs. <20%)	-	0.68 (0.55–0.84)	0.74 (0.57–0.95)	0.65 (0.53–0.79)	0.65 (0.55–0.76)	0.68 (0.55–0.84)	0.72 (0.57–0.91)	0.67 (0.54–0.83)	0.69 (0.56–0.85)

IOR	-	-	-	-	-	-	-	-	-

**Random Effects**									

**ZIP5 **[Var. (SE)]	0.513 (0.165)	0.505 (0.155)	0.467 (0.134)	0.518 (0.159)	0.487 (0.130)	0.501 (0.150)	0.495 (0.152)	0.506 (0.155)	0.462 (0.122)

MOR^†^	1.98	1.97	1.92	1.99	1.95	1.96	1.96	1.97	1.91

ICC (%)	13.49	13.31	12.43	13.60	12.89	13.22	13.08	13.33	12.31

PCV^‡ ^(%)	**-**	-1.56	-8.97	-0.97	-5.07	-2.34	-3.51	-1.36	-9.94

**ZIP3 **[Var. (SE)]	0.095 (0.033)	0.016 (0.020)	0	0.021 (0.022)	0	0.013 (0.018)	0.019 (0.020)	0.017 (0.020)	0

MOR	1.34	1.13	1.00	1.15	1.00	1.11	1.14	1.13	1.00

ICC (%)	2.81	0.48	0	0.63	0	0.39	0.57	0.51	0

**ZIP3 & ZIP5**	0.608	0.521	0.467	0.539	0.487	0.514	0.514	0.523	0.462

MOR	2.10	1.99	1.92	2.01	1.95	1.98	1.98	1.99	1.91

**DIC**^§^	4213	4151	4056	4119	3747	4145	4125	4142	3659

Note: Model I is an empty model; Model II only contains area poverty; Model III contains area poverty and demographic variable (age, gender, age, race/ethnicity, marital status, education, employment and income); Model IV contains area poverty and health condition variables (BMI < weight/height^2^>, chronic diseases history <asthma, diabetes, stroke, coronary heart disease and heart attack> and depression/anxiety history); Model V contains area poverty and access to care variables (health plan coverage, primary care physician and routine check-up for medical status); Model VI contains area poverty and emotional support; Model VII contains area poverty and health-related behaviors (smoking, alcohol-consumption and physical exercise); Model VIII contains area poverty and self-rated health status; Model IX contains area poverty and all individual-level factors. * IOR: Interval odds ratio; ^† ^MOR: Median odds ratio; ^‡ ^PCV: Proportional change in variance relative to Model I; §DIC, Deviance information criterion; ^¶ ^*P *< 0.01.

**Table 5:** Association between FOBT use and poverty rate at ZIP5 and ZIP3 level by examining six potential pathways.

	Model I	Model II	Model III	Model IV	Model V	Model VI	Model VII	Model VIII	Model IX
**Fixed Effects**									

**ZIP5 poverty **(>= 10% vs. <10%)	-	1.21 (0.85–1.72)	1.28 (0.90–1.82)	1.22 (0.85–1.74)	1.25 (0.87–1.80)	1.21 (0.85–1.71)	1.24 (0.87–1.75)	1.21 (0.85–1.73)	1.37 (0.95–1.97)

IOR *	-	0.21–6.97	0.20–8.09	0.20–7.29	0.22–7.08	0.20–7.23	0.21–7.15	0.21–7.04	0.20–9.29

**ZIP3 poverty **(>= 20% vs. <20%)	-	0.83 (0.64–1.07)	0.83 (0.62–1.11)	0.85 (0.66–1.11)	0.79 (0.58–1.06)	0.81 (0.62–1.05)	0.84 (0.65–1.09)	0.83 (0.64–1.07)	0.76 (0.54–1.07)

IOR	-	-	-	-	-	-	-	-	-

**Random Effects**									

**ZIP5 **[Var. (SE)]	0.910 (0.253)	0.933 (0.262)	1.035 (0.289)	0.977 (0.263)	0.914 (0.273)	0.976 (0.271)	0.937 (0.265)	0.940 (0.264)	1.117 (0.317)

MOR^†^	2.48	2.51	2.64	2.57	2.49	2.57	2.52	2.52	2.74

ICC (%)	21.67	22.09	23.93	22.90	21.74	22.88	22.17	22.22	25.35

PCV^‡ ^(%)	**-**	2.53	13.74	7.36	0.44	7.25	2.97	3.30	22.75

**ZIP3 **[Var. (SE)]	0.116 (0.075)	0.114 (0.074)	0.111 (0.084)	0.114 (0.077)	0.151 (0.094)	0.110 (0.073)	0.111 (0.073)	0.116 (0.075)	0.146 (0.104)

MOR	1.38	1.38	1.37	1.38	1.45	1.37	1.37	1.38	1.44

ICC (%)	3.41	3.35	3.26	3.35	4.39	3.24	3.26	3.41	4.25

**ZIP3 & ZIP5**	1.026	1.047	1.146	1.091	1.065	1.086	1.048	1.056	1.263

MOR	2.63	2.65	2.78	2.71	2.68	2.70	2.66	2.67	2.92

**DIC**^§^	1323	1313	1190	1303	793	1283	1268	1313	713

Note: Model I is an empty model; Model II only contains area poverty; Model III contains area poverty and demographic variable (age, gender, age, race/ethnicity, marital status, education, employment and income); Model IV contains area poverty and health condition variables (BMI < weight/height^2^>, chronic diseases history <asthma, diabetes, stroke, coronary heart disease and heart attack> and depression/anxiety history); Model V contains area poverty and access to care variables (health plan coverage, primary care physician and routine check-up for medical status); Model VI contains area poverty and emotional support; Model VII contains area poverty and health-related behaviors (smoking, alcohol-consumption and physical exercise); Model VIII contains area poverty and self-rated health status; Model IX contains area poverty and all individual-level factors. * IOR: Interval odds ratio; ^† ^MOR: Median odds ratio; ^‡ ^PCV: Proportional change in variance relative to Model I; §DIC, Deviance information criterion; ^¶ ^*P *< 0.01.

## Discussion

Our previous study showed that only 1.2% of the variation in CRC screening existed among metropolitan or micropolitan statistical areas across the United States, which are urban areas with populations of at least 10,000 residents [17]. In contrast, nearly 15 percent of the CRC screening variation was across ZIP5 areas (MORs ≈ 2) in Missouri. No variation existed across ZIP3 areas in the current study. Thus, larger variation in CRC screening appears to exist among smaller geographic areas within one state, not among large geographic areas across the United States. It is possible that the disparity pattern is different in Missouri compared with the disparity in CRC screening nationwide, but additional studies are needed to examine this.

In addition, CRC screening use was found to be significantly lower in high-poverty areas than in low-poverty areas in Missouri independent of the individual-level factors included in this study. In a previous study, we also found an independent association between metropolitan or micropolitan statistical area-level poverty rates and CRC screening across U.S. communities using the 2002 BRFSS data [17]. Our current study builds on these findings by considering additional individual-level factors, including health condition, access to care, social support, health-related health behaviors and self-rated health status, but an independent effect of area-level poverty remained. This suggests that the hypothesized factors did not mediate the association between area-level poverty and CRC screening. Since IOR values contained the value of one, this implies area-level poverty did not account for much of the geographic variation of CRC screening in Missouri. For persons residing within the same areas, those who were a non-black minority, had a lower level of education, mid-low levels of family income and lack of access to medical care had lower odds of CRC screening. Conversely, those who were retired and those with a higher BMI and anxiety/depression history had higher odds of CRC screening. These findings are similar to other studies that examined only individual level variables [11,14,18].

While CRC screening has been increasing over time, this is largely due to increasing colonoscopy use. Because of the higher cost associated with this test relative to FOBT, low-income persons, persons without health insurance, or those who live in high poverty areas may be especially affected. Our results show this since area-level poverty was associated with endoscopy use but not FOBT use. As a result, disparities between population subgroups may increase over time if colonoscopy becomes the predominant CRC screening test.

In our study, area-level poverty did not account for much of the geographic variation of CRC screening. Also, the effect of area-level poverty rate on CRC screening was not fully explained by individual-level factors included. Therefore, additional area-level and individual-level factors need to be identified that can explain the geographic variation of CRC screening. Other area-level and individual-level factors not included in the BRFSS, such as managed care coverage, availability of primary care physicians, knowledge about cancer risk factors, psychosocial factors, and physician recommendations could play a role in the association between adverse area poverty and CRC screening [21,28,29]. In addition, availability and accessibility of health care services (including safety-net clinics) [8] may result in the lack of access to medical care because of a possible travel barrier.

One of the frequent criticisms of multilevel models using geographic areas is that in some cases the choice of the geographic unit is somewhat arbitrary and may not reflect meaningful neighborhoods or communities. In our study, we used self-reported ZIP codes of participants' residency as the geographic unit. While ZIP codes cannot be considered neighborhoods or communities, it appears that the variation in CRC screening is large and that poverty rate exerts an important effect on CRC screening at this level of aggregation. We used the zip code tabulate area (ZCTA5) from the 2000 census to estimate the ZIP code poverty. ZCTA5 was developed by the U.S. Census Bureau and is a close approximation to self-reported ZIP code. Because ZCTA5 areas are not an exact geographic match to ZIP code areas, mismatching bias may exist [42]. We used ZIP3 as the third geographic level, which decreased the mismatching bias to some degree because it is a larger geographic area than ZIP5 areas. BRFSS questions do not ask about the interviewee's street address, thereby precluding analysis at other spatial scales. Although the BRFSS contains a question inquiring about the county of residence, counties are much larger in size than census block groups or tracts. Using county as area-level unit will ignore the intracounty variation that appears to exist at the ZIP5 level, although ZIP5 areas may cross county borders.

Limitations of this study include our reliance on self-reported CRC screening use, coverage bias in traditional telephone surveys of low-income population, and a relative low response rate of the BRFSS survey (58.5%). However, self-reported CRC screening was similar to medical record data [47]. In recent years, the proportion of adults with only wireless telephones is growing rapidly in the United States. In 2006, 17 percent of low-income adults with household income below 200 percent of the federal poverty thresholds lived in a household with only cell telephones [48]. Only using land lines as sampling frame was unlikely to bias our results since less than three percent of persons age 55 or older have only a cell phone [49]. Even though BRFSS post-stratification weights offset coverage bias and non-response bias to some degree [50], this may still results in an underestimation of CRC screening, especially in ZIP5 areas with higher poverty rate. This may also lead to the underestimation of geographic variations of CRC screening and the effect of area-level poverty rates on CRC screening. Despite this, we found substantial geographical variation of CRC screening across ZIP5 areas in Missouri and significant associations between CRC screening and area-level poverty rates. Additionally, in this study we used a combined measure of CRC screening adherence. Since factors associated with FOBT and endoscopy use may differ in Missouri, future studies should consider examining the individual and geographic disparities in FOBT and endoscopy use in order to maximize the effectiveness of future interventions.

We expect that our results may have been underestimated by not taking into account the length of residence in the same house or area. In our other work in the St. Louis area, we found that the adverse effect of neighborhood characteristics on disability and diabetes incidence was more pronounced when limiting the study population to persons who have lived in the same house for at least 5 years [51,52].

Although our study population was restricted to Missouri residents and formal generalizability will be only to those residents, our study population is in many ways similar to the entire U.S. population in terms of its sociodemographic characteristics. The St. Louis area in Missouri is one of only a few CDC-funded programs aimed at screening low-income and underinsured persons for CRC. Despite this program, our results show that persons who live in areas with elevated poverty were less likely to be screened for CRC.

## Conclusion

CRC screening varies across ZIP codes in Missouri. Area-level poverty rates are associated with CRC screening and partly explain the geographic variation of CRC screening. Individuals residing in higher-poverty areas have lower odds of CRC screening independent of the individual-level factors included in the model. It suggests that area-level poverty rates can be considered an important area characteristic for targeting interventions related to CRC screening, especially for endoscopy use. However, additional research is needed to identify other area characteristics to further explain the remaining area heterogeneity in CRC screening. To maximize potential intervention effectiveness in areas of elevated poverty, additional individual-level factors should be examined as part of the pathways through which adverse area environments exert their influence on CRC screening.

## Abbreviations

Abbreviations applied in the text and tables include: CRC: colorectal cancer; BRFSS: Behavioral Risk Factor Surveillance System; ZIP5: five-digit ZIP code; ZIP3: three-digit ZIP code; OR: Odds ratio; CI: confidence interval; FOBT: fecal occult blood test; ACS: American Cancer Society's; ZCTA5: 5-digit Zip Code Tabulate Area; ZCTA3: 3-digit Zip Code Tabulate Area; BMI: body mass index; PQL: penalized quasi-likelihood; DIC: deviance information criterion; ICC: intraclass correlations; MOR: median odds ratio; IOR: interval odds ratio.

## Competing interests

The authors declare that they have no competing interests.

## Authors' contributions

ML designed the study, performed data analysis and wrote the manuscript; MS helped design the study and wrote sections of the manuscript; SY prepared the original data and helped write the manuscript. All authors have read and approved the final version of the manuscript.

## Pre-publication history

The pre-publication history for this paper can be accessed here:


